# Automated 3D bio-imaging analysis of nuclear organization by NucleusJ 2.0

**DOI:** 10.1080/19491034.2020.1845012

**Published:** 2020-11-29

**Authors:** Tristan Dubos, Axel Poulet, Céline Gonthier-Gueret, Guillaume Mougeot, Emmanuel Vanrobays, Yanru Li, Sylvie Tutois, Emilie Pery, Frédéric Chausse, Aline V. Probst, Christophe Tatout, Sophie Desset

**Affiliations:** a GReD, CNRS, INSERM, Université Clermont Auvergne, Clermont-Ferrand, France58; b Department of Molecular, Cellular & Developmental Biology, Yale University, New Haven, CT, USA; c Neurodol, INSERM, Université Clermont Auvergne, Clermont-Ferrand, France; d Department of Biological and Medical Sciences, Faculty of Health and Life Sciences, Oxford Brookes University, Oxford, UK; e Department of Plant and Microbial Biology, Zürich-Basel Plant Science Center, University of Zürich, Zürich, Switzerland; f Institut Pascal, Université Clermont Auvergne, Clermont-Ferrand, France

**Keywords:** Three-dimensional microscopy imaging, image analysis, plant nucleus, nuclear organization, 3D DNA FISH

## Abstract

NucleusJ 1.0, an ImageJ plugin, is a useful tool to analyze nuclear morphology and chromatin organization in plant and animal cells. NucleusJ 2.0 is a new release of NucleusJ, in which image processing is achieved more quickly using a command-lineuser interface. Starting with large collection of 3D nuclei, segmentation can be performed by the previously developed Otsu-modified method or by a new 3D gift-wrapping method, taking better account of nuclear indentations and unstained nucleoli. These two complementary methods are compared for their accuracy by using three types of datasets available to the community at https://www.brookes.ac.uk/indepth/images/. Finally, NucleusJ 2.0 was evaluated using original plant genetic material by assessing its efficiency on nuclei stained with DNA dyes or after 3D-DNA Fluorescence *in situ* hybridization. With these improvements, NucleusJ 2.0 permits the generation of large user-curated datasets that will be useful for software benchmarking or to train convolution neural networks.

## Introduction

Investigation of nuclear morphology and its impact on chromatin organization is an active field [1]. Identification of key players that determine nuclear morphology at the nuclear periphery is a complex task, for which the model plant *Arabidopsis thaliana* offers an amenable genetic system. As in animals, the plant nucleus is delimited by a double membrane interrupted by nuclear pores, which allow exchanges with the cytoplasm. The outer nuclear membrane (ONM) is connected to the endoplasmic reticulum and the cytoskeleton, while the inner nuclear membrane (INM) is linked with a filamentous network constituting the nucleoskeleton [2]. The Linker of Nucleoskeleton and Cytoskeleton (LINC) complex provides a junction between the interior of the nucleus and the cytoplasm by means of SUN (Sad1 and Unc-84 homology) proteins anchored in the INM that interact with the nucleoskeleton [3,4] and KASH (Klarsicht, Anc-1 and Syne homology) proteins anchored in the ONM and connected to the cytoskeleton, respectively [5,6]. In animals, the nucleoskeleton is made of lamins. Plants do not have lamin orthologs in term of sequence homology, but the CROWDED NUCLEI (CRWN) proteins, which contain long coiled-coil regions like lamins, are likely to have similar functions in nuclear morphology and chromatin organization [7,8] as well as in regulation of gene transcription [9,10]. Other proteins are thought to be involved in the constitution, or anchoring, of the nucleoskeleton at the nuclear periphery, such as KAKU4 [11] and NUCLEAR ENVELOPE ASSOCIATED PROTEINS (NEAPs) [12]. Most of these protein components have been shown to impact nuclear morphology, nuclei becoming usually smaller and more spherical in mutant backgrounds [13], allowing genetic screens for mutants with altered nuclear morphology [11]. To better understand the impact of the nuclear periphery on nuclear organization, molecular techniques applied to the whole genome such as chromatin conformation capture (Hi-C) or Chromatin Immunoprecipitation (ChIP-Seq) have been applied in plants and brought a new vision of the 3D genome [14–16].

Bio-imaging is a complementary approach, in particular when coupled to quantitative image analysis [17]. However, the throughput of bio-imaging limits its routine use in 3D image processing [18]. To this aim, more automated methods are needed and efficient 3D segmentation is required to delimit the boundary of objects such as the nucleus or its chromatin organization [19]. Investigation of the cell nucleus has strongly benefited from these applications, as the nucleus is a spatial structure for which morphology can be modified in diseases [20,21]. Segmentation applied to nuclear organization is also a research tool to investigate the genetic determinants of nuclear size and shape and also chromatin organization [22,23]. There are various softwares packages dedicated to nuclear segmentation [24]. These are usually developed for the detection of nuclei in a wide-field stack and to compute morphology parameters, but only a few of these go as far as the analysis of the content and organization of chromatin [25,26]. Moreover, most of them are optimized for one given tissue or cell type but they are not functional on images containing a large diversity of nuclear size and shape as found in plants [27]. These limitations motivated the development of an ImageJ plugin called NucleusJ dedicated to the analysis of nuclear organization of 3D plant nuclei [23]. Within NucleusJ 1.0, segmentation methods to compute nuclear morphology (a modified Otsu threshold method) and chromatin organization (a 3D watershed method) were chosen as the most relevant methods for nuclear segmentation for 3D nuclei. Although initially developed for plant nuclei stained with DNA dyes [23,28,29], NucleusJ 1.0 can also be used for other cell types [20] and adapted to segment Fluorescence *in situ* hybridization (FISH) signals [29]. However, each NucleusJ 1.0 analysis is time-consuming, the segmentation threshold is user-dependent and nuclear segmentation failed for a substantial fraction of nuclei.

Here, we introduce the optimized NucleusJ version termed NucleusJ 2.0. To increase the number of nuclei considered in a single analysis, a method was introduced to delimit an automatic bounding volume (autocrop) around each nucleus of a 3D wide-field stack containing 10 to a hundred nuclei. Each of the collected nuclei can then be segmented through two complementary methods, either based on the Otsu threshold method or on edge-detection through a 3D gift-wrapping method. From the segmented objects, NucleusJ 2.0 computes new nuclear morphology parameters using a revised and more accurate method of nuclear surface calculation. The accuracy of the measurements performed with NucleusJ 2.0 was confirmed with digitized spheres and multicolor fluorescent beads of standardized sizes. NucleusJ 2.0 was then used to characterize nuclei stained with DNA dyes or labeled with 3D-DNA FISH in whole-mount tissue of a plant mutant with strong alteration of nuclear morphology and chromatin organization. Finally, computation efficiency of NucleusJ 2.0 has been optimized to include a command line version that can be used on distant servers at computing centers.

## Material and methods

### Plant materials

**Figure 1.**
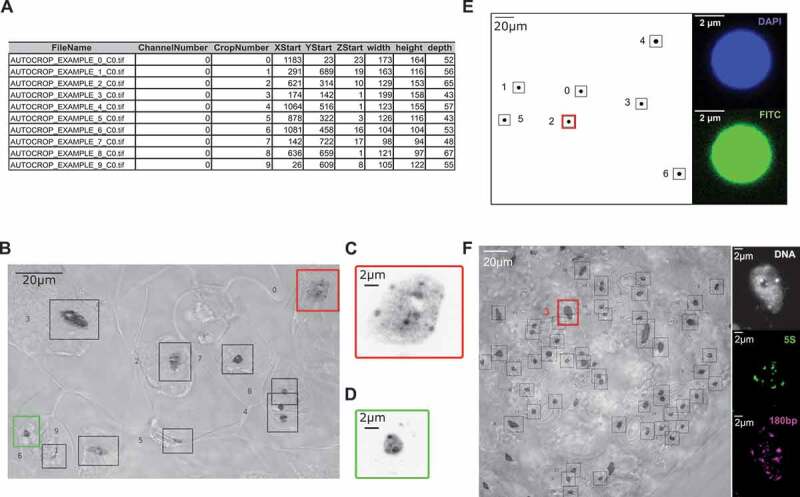
Application of NucleusJ 2.0 autocrop method on a wide-field stack

All mutant and wild type (WT) *Arabidopsis thaliana* plants were from the Columbia-0 (Col-0) ecotype. Mutant lines were T-DNA insertions obtained from The Nottingham Arabidopsis Stock Center (NASC): *kaku4-2* (SALK_076754), *crwn1-2* (SALK_041774) and *crwn4-1* (SALK_079296). The triple mutant *kaku4-2 crwn1-2 crwn4-1* (*k4c1c4*) was obtained by genetic crosses. Cotyledons for image acquisitions were grown from sterilized seeds sown on germination medium containing 0.8% w/v agar, 1% w/v sucrose and Murashige & Skoog salts (M0255; Duchefa Biochemie, Netherlands), grown at 23°C and harvested 13 days after germination (dag). For phenotypic evaluation, seedlings were grown on soil in an Arabilab growth chamber at 20°C. In both cases, seeds were subjected to 2 days of stratification at 4ºC in the dark and then grown under 16 h light/8 h dark cycles. The leaf area of the 21-day-old plants was determined with the ImageJ software using the SIOX (Simple Interaction Object eXtraction) plugin [30]. 13 dag cotyledons were used to determine the number of stomates, guard cell, and pavement cell nuclei. To this aim a maximum Z-projection of a wide-field stack stained with Hoechst 33,258 and a single plane image under transmission light using Differential Interference Contrast (DIC) were combined (overlay) as described in Figure 1.

(a) Output table from a typical experiment ‘Autocrop_example’ using Arabidopsis cotyledons stained with Hoechst 33,258 DNA dye.(b) Overlay of a maximum Z-projection of the Autocrop_example wide-field stack gained from a sample of cotyledon stained with Hoechst 33,258 and a single plane image under transmission light using Differential Interference Contrast (DIC). Coordinates (X, Y and Z start) of the nine nuclei are described in Figure 1a. DIC allows identifying nuclei of guard cells (autocrop boxes #0 and # 6, respectively, bordered in red and green), which are located at stomates and pavement cells outside of stomates. Autocrop automatically draws bounding boxes (from 0 to n) of the overlay and inverted Lookup Table (LUT) to easily look at the nuclei position. This example also highlights rare cases where the bounding box contains two different nuclei (autocrop box #4).(c) and d) are close-up images of sub-regions of the Z-projection illustrating, respectively, a large pavement cell nucleus (autocrop box #0 in red) and a smaller and rounder guard cell nucleus (autocrop box #6 in green). (e) Multi-channel autocrop on 4 µm microspheres. Maximum Z-projection of a wide-field stack (left) with seven microspheres (#0 to 6). Selected Z-slice (right) of the same microsphere (autocrop box#2 in red) in two fluorescent channels; DAPI channel (top), FITC channel (bottom).(f) Multi-channel autocrop on plant nuclei. Overlay of a maximum Z-projection of the autocrop in DAPI channel wide-field stack (left) with 46 nuclei. Selected Z-slice (right) of nucleus #3 (red box) in three fluorescent channels; DAPI channel (gray level), Cy5 for 5S rDNA probe (green level) and Cy3 for 180bp probe (magenta level).

### Digitized spheres and microspheres

Digitized spheres of various radii of 5, 10, 20, 30, 40, and 50 voxels were designed as binary objects (0 for the background, 1 for the object) using isotropic object voxels (*i.e*. cubes where X, Y, Z-axis values are 1, 1, 1) and background voxels that do not belong to the object (Supplemental table 1). Fluorescent microspheres are standardized polystyrene beads commonly used for alignment and calibration of confocal microscopes. Slides containing fluorescent microspheres of 1, 2.5, and 4 µm diameter (Invitrogen) were used in this study (Supplemental table 2).

**Table 1.** OMERO-FSU Datasets

DATASET NAMES	ACQUISITION SYSTEM	DATASET NAMES	LINK	KEY FILTER	VALUE FILTER	
#1 DIGITIZED SPHERES	Wide-field + OptoGrid	#1a DIGITIZED SPHERES – RAW	dataset-4902	RAW	DIGITIZED	6
		#1b DIGITIZED SPHERES – SEGMENTED	dataset-4901	SEGMENTATION	OTSU	6
					GIFT-WRAPPING	6
#2 FLUORESCENT MICROSPHERES	Wide-field + OptoGrid	#2a FLUORESCENT MICROSPHERES – ACQUISITION	dataset-4903	RAW	WIDE FIELD STACK	17
		#2b FLUORESCENT MICROSPHERES – AUTOCROP	dataset-4951	PICTURE TYPE	Z MAX PROJECTION	17
		#2 c FLUORESCENT MICROSPHERES – RAW	dataset-4952	RAW	DAPI CROP	81
					FITC CROP	97
		#2d FLUORESCENT MICROSPHERES – SEGMENTED	dataset-4953	SEGMENTATION	OTSU	157
					GIFT-WRAPPING	157
#3 NUCLEAR MORPHOLOGY	Wide-field + OptoGrid	#3a NUCLEAR MORPHOLOGY – ACQUISITION	dataset-4954	RAW	WIDE FIELD STACK	63
		#3b NUCLEAR MORPHOLOGY – AUTOCROP	dataset-4955	PICTURE TYPE	DIC	63
					Z MAX PROJECTION	63
					OVERLAY	63
		#3 c NUCLEAR MORPHOLOGY – RAW	dataset-4956	RAW	DAPI CROP	1554
		#3d NUCLEAR MORPHOLOGY – SEGMENTED	dataset-4957	SEGMENTATION	OTSU	1554
					GIFT-WRAPPING	1553
		#3e NUCLEAR MORPHOLOGY – RAW BAD CROPS	dataset-5009	RAW	BAD CROP	102
#4 CHROMATIN ORGANIZATION	Wide-field + OptoGrid	#4 CHROMATIN ORGANIZATION – RAW	dataset-5018	SEGMENTATION	THRESHOLD WATERSHED	315
#5 180BP_5S DNA FISH	Wide-field + OptoGrid	#5a 180BP_5S DNA FISH – ACQUISITION	dataset-5010	RAW	WIDE FIELD HYPERSTACK	8
		#5b 180BP_5S DNA FISH – AUTOCROP	dataset-5011	PICTURE TYPE	DIC	4
					Z MAX PROJECTION	4
					OVERLAY	4
		#5 c 180BP_5S DNA FISH – RAW	dataset-5012	RAW	CY3 CROP	160
					CY5 CROP	149
					DAPI CROP	160
		#5d 180BP_5S DNA FISH – GIFT SEGMENTED	dataset-5015	SEGMENTATION	GIFT-WRAPPING	159
		#5e 180BP_5S DNA FISH – FISH SIGNALS	dataset-5016	SEGMENTATION	CY3 THRESHOLD WATERSHED	129
					CY5 THRESHOLD WATERSHED	159
#6 5S DNA FISH	Confocal	#6a SINGLE DNA FISH – RAW	dataset-5019	RAW	DAPI CROP	80
					CY3 CROP	80
		#6b SINGLE DNA FISH – GIFT SEGMENTED	dataset-5021	SEGMENTATION	GIFT WRAPPING	80
		#6 c SINGLE DNA FISH – FISH SIGNALS	dataset-5022	SEGMENTATION	CY3 THRESHOLD WATERSHED	75

Six types of image datasets (Dataset names) gained from wide-field or confocal microscopy (Acquisition systems) were stored as six main directories at OMERO-FSU under the name IDP3006_Dubos-Desset_2020. For each dataset, images were organized in acquisition, autocrop, raw and segmented sub-directories and can be directly accessed using the web link included into Table 1 (Link). OMERO allows to screen for key-value pairs (Key and value filters). Number of images are indicated in the last column. The six datasets and their image processing represent a total of 7,313 images.

### Sample preparation, DNA staining and 3D DNA-FISH

3D images were obtained from whole mount preparations of 13 dag cotyledons as previously described [29,31]. Whole mount preparations were then used either for DNA staining using Hoechst-staining procedure (Hoechst 33 258 overnight at 1 µg/ml final) or for 3D-DNA Fluorescence *In Situ* Hybridization (3D DNA FISH). 3D DNA FISH experiments were performed as previously described [32]. 5S rDNA probe was produced from pCT4.2 vector [33] amplified with 5ʹCY5-dUTP and directly-labeled oligonucleotides (/CY5/CCCAAATTTTGACCTTTAAG) and (/CY5/GTCGACAAAAAGTCAATGGA) or with 5’CY3-dUTP and the same oligonucleotides without fluorescent labels. 180pb satellite repeat probe was designed as an LNA-oligonucleotide (/5TYE563/GTATGATTGAGTATAAGAACTTAAACCG – Qiagen) [34].

### Microscope and image acquisition

Microscopic observations were performed by structured illumination microscopy to produce wide-field stacks using an Optigrid module (Leica-microsystems MAAF DM 16000B). All stacks were acquired using an X63 oil N.A. 1.4 objective and a digital CMOS Camera (ORCA-Flash4.0 V2 C11440-22 CU – Hamamatsu) at an optimal resolution such that lateral and axial resolution were respectively XY = 0.103 µm and Z = 0.2 µm. For better resolution, some confocal images were acquired with a Zeiss LSM 800 with an X63 oil N.A. 1.4 objective and a voxel size XY = 0.60 µm and Z = 0.2 µm. Final image numbers are given for each dataset in Table 1.

### Datasets storage and availability

Datasets were stored at OMERO-Florida State University (OMERO-FSU), a public repository under the accession number IDP3006 Dubos–Desset Nucleus 2020 that can be accessed through the INDEPTH COST-Action (CA16212) website at https://www.brookes.ac.uk/indepth/images/. The INDEPTH image webpage provides a guideline to access and download the datasets that are freely available for research purposes. More tutorials to use OMERO are available at the OMERO webpage at https://www.openmicroscopy.org/.

Six types of datasets were produced for this study (#1 to #6) (Table 1). Each dataset was stored at OMERO-FSU under the accession number IDP3006_Dubos-Desset_2020 where it was organized in four folders: acquisition (wide-field stacks or confocal images), autocrop (results of autocrop processing), raw (3D images containing a single nucleus per stack) and segmented (raw image after segmentation) except for dataset#1, which was generated *in silico* and for that reason no acquisition nor autocrop was performed. OMERO allows screening for a subset of each dataset using key-pair values as described in Table 1. A training dataset is also available at OMERO-FSU under the accession number IDP2002 Dubos – Desset 2020.

### NucleusJ 2.0 algorithm development

Documentation: NucleusJ 2.0 is an ImageJ plugin in Java language released as a jar file for the ImageJ platform. Installation and usage guides are available at https://gitlab.com/DesTristus/NucleusJ2.0. The software contains a 3D autocrop module and three independent segmentation methods hereafter referred to as the modified Otsu, 3D gift-wrapping and 3D watershed methods. NucleusJ was described in a previous publication [23]. New functionalities or improvements are detailed below.

Autocrop: From any given 2D or 3D wide-field image a simple Otsu threshold [35] was applied to obtain a binary image *i.e*. transforming all the voxels of the image to a value of 0 for the background voxels and 1 for each voxel from the object (nucleus). Volume of each connected voxel (*i.e*. connected component) was computed using the MorpholibJ library [36] and connected components above 1 µm were conserved. Finally, for each connected component, a coordinate box was designed by adding as a default parameter 20 voxels at the most extreme voxels (*i.e*. boundary voxels) coordinated in each X, Y and Z dimensions. Note that the number of added voxels and hence the size of the coordinate box can be modified according to the specific application. An optional and configurable step is available when multiple coordinate boxes are produced for a single nucleus. This step groups boxes when they display a 50% shared surface. Finally, a maximum Z-projection of the initial wide-field image was automatically generated as a.*tif* file. In this 2D image, each nucleus was numbered and surrounded by its coordinate box. A tabulated file containing the list of nuclei and coordinate boxes was produced as a .*txt* file. Documentation is provided as Supplemental file 1.

Gift-wrapping: To provide an independent and complementary method to the modified Otsu threshold method available in NucleusJ 1.0, the Jarvis march algorithm [37] was implemented into NucleusJ 2.0 (Supplemental file 2). For the sake of simplicity, the method was designed in 2D and implemented slice by slice to the whole 3D object. Hence, three axes *i.e*. XY, XZ, and YZ were used to decompose the 3D volume in 2D slices. The method then computed the union of each possible plan in XY XZ YZ. For each slice, in order to tune the 3D gift-wrapping algorithm, and to fill the shape artifacts as well as possible, a parameter of maximal threshold distance *td* was applied between two vertices defining the final boundary. The best threshold distance was determined experimentally as the half of the estimated radius of a sphere with a volume equivalent to the object one (Supplemental file 2).

SurfaceArea calculation: When analyzing 3D objects, the surface area plays an important role for shape description. In NucleusJ 1.0, the surface area parameter was computed for any given object as the sum of all voxel boundary exposed to the background. This was improved in NucleusJ 2.0 to better take into account the contribution of each surface element area using the discrete geometry technique [38]. The first step of the area calculation was to determine the image gradient of the raw image f, which was estimated from finite differences in the anisotropic image (Supplemental file 3). The algorithm then browsed each boundary voxel of the segmented object. For each boundary voxel, the contribution of each surface element area was then computed for the final area.

Nuclear morphology parameters in NucleusJ 2.0: NucleusJ 2.0 was revisited for its functionalities to segment nuclei. Description of the quantitative parameters generated by NucleusJ 1.0 can be found in supplemental materials of [23]. Parameters computed by NucleusJ 2.0 are described in Supplemental file 4.

### Statistics

Statistical analyses were performed using various R packages [39] for Principal Component Analysis (PCA) ggplot2 [40], factoextra (https://rpkgs.datanovia.com/factoextra/) and FactoMineR [41] were used. Student t-test was used to compare means between WT and mutant backgrounds.

## Results

### Automatic selection of 3D nuclei from wide-field stacks using a 3D autocrop process

Any kind of 3D bio-image analysis starts with the capture of images of best quality. During this initial step, 3D stacks containing the objects of interest, such as a cell nucleus, are collected either one by one when using a confocal microscope or by collecting multiple nuclei at a time using wide-field microscopy. In the latter, bounding boxes surrounding the 3D nuclei have to be defined to delimit and extract (crop) the appropriate volume containing each nucleus. Automatic tracking of segmented objects would strongly reduce this tedious manual step. Setting-up such middle to high-throughput 3D tracking process is a timely objective when using wide-field stacks to rapidly buildup large image datasets [42].

While ImageJ offers 3D crop and 2D autocrop plugins [42,43] automated and scalable 3D autocrop plugins to extract multiple nuclei from wide-field stacks is missing. Furthermore, detection of multiple objects should take into account variable fluorescence intensity between objects that does not allow the same segmentation threshold to be applied to the whole stack.

Our initial motivation was to implement a simple and rapid method to automatically identify and isolate large numbers of 3D nuclei from wide-field stacks, a process hereafter called autocrop. Once the objects are delimited in the wide-field stack, more sophisticated segmentation methods could be applied for each single object regardless of their fluorescence intensity. The autocrop basic principle relies on a simple Otsu threshold method [35] applied in 3D to the wide-field stack (Materials and Methods). To avoid the selection of too many objects from the background, a simple and scalable size filter was introduced. In our plant model, this has been useful to filter-out chloroplasts that as autofluorescent objects are considered as noise in our analyses. A second scalable filter allows limiting the number of multiple boxes for a given object, which was proven to be helpful especially for large nuclei. Default autocrop parameters can be modified prior to the analysis through a simple configuration file (*config file*). More details are available in the autocrop documentation (Supplemental file 1). The method is illustrated below for 3D stacks although it can also be used for 2D images.

The autocrop process with default settings was applied for microspheres and wide-field stacks of whole mount cotyledons of WT stained with DNA dyes (Materials and Methods; Supplemental tables 2–3). The autocrop produces i) a collection of 3D images containing a single object stored in a dedicated folder, ii) a table containing the spatial coordinates of the bounding box (X, Y and Z) to trace back their positions in the original wide-field stack, iii) the bounding box volumes (width, height, and depth) and iv) an inverted color 2D Z-projection using maximum intensity projection, in which each object is numbered (Figure 1(a-c)). The number of cropped objects obtained after the autocrop process depends on the density and size of the objects within the original wide-field stack. For instance, 91 successful crops were obtained from 3 wide-field images for 1 µm microspheres, while only 46 crops were obtained for 4 µm microspheres (Supplemental table 2). When imaging the epidermis of an Arabidopsis cotyledon, a typical experiment described in Supplemental table 3 that starts with 12 WT plants generates 35 wide-field stacks (average of 3 images per cotyledon) allows generating 786 crops using the autocrop method (average of 22 crops per wide-field stack). Once the autocrop is performed, an overlay of the Z-projection allows tracing back the nuclei in their tissue context and annotating them regarding their origin (*i.e*. guard cell or pavement cell nuclei) or their pertinence (some objects that are not nuclei are detected) (Figure 1(b-d)). In this experiment, the Z-projection inspection allowed discarding 92 abnormal nuclei. Finally, 694 nuclei (88% of the initial crops) were usable to further analysis of their nuclear morphology.

An interesting functionality of the autocrop is to generate multiple autocrops from a given wide-field stack using the same coordinate table to select identical boxes in different wavelength channels. This application is illustrated with microspheres of 4 µm labeled with fluorophores emitting in the DAPI (blue) and FITC (green) channels (Figure 1(e)) and with plant nuclei labeled with DNA dyes and two fluorescent probes emitting in two distinct channels (figure 1(f)). This functionality allows capturing DAPI and probe channels in typical Fluorescence *in situ* hybridization (FISH) experiments from the same image.

In our hands, the 3D autocrop method applied to wide-field stacks is an efficient automated process to detect position and isolate large numbers of nuclei that can then be subjected to further image analysis.

### 3D gift-wrapping as a complementary method for nuclear segmentation of plant nuclei

**Figure 2.**
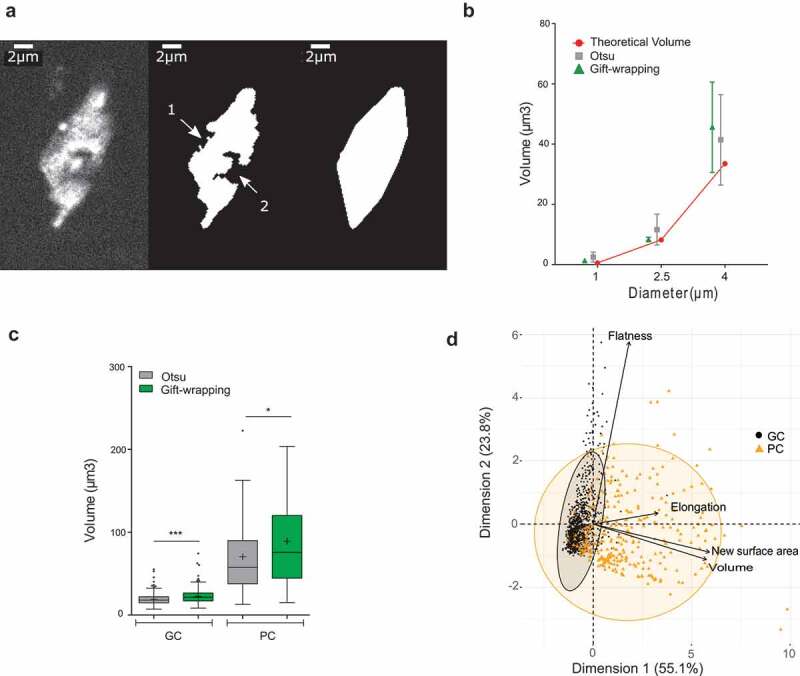
Evaluation of a 3D gift-wrapping method of segmentation

There is an increasing demand in biology to describe nuclear morphology (shape and size) or to evaluate the organization of chromatin domains [18,44]. A first step in such an image analysis is to delimit the nucleus from the background, a process called segmentation. We previously developed a modified Otsu threshold segmentation process delimiting the nuclear boundaries according to their range of gray scale values [23]. However, despite the improvement of the original Otsu method, 10–20% of nuclei with poor segmentation still needed to be discarded after manual curation of the images (Figure 2(a)). Standardization of sample preparation and image acquisition protocol [31] did not overcome this bias.

(a) Example of a nucleus raw slice (left) after Otsu-modified segmentation (middle) and gift-wrapping segmentation (right). Artefactual indentation at the nuclear border (arrow #1); Nucleolus border (arrow #2).(b) Comparison of Otsu and gift–wrapping methods using standardized microspheres. Microsphere volume of 1, 2.5 and 4 µm diameter (n = 28, 24, and 15, respectively) were computed by the Otsu (green triangle) and gift-wrapping (gray square) methods and compared to theoretical volumes (red circle) (Supplemental table 2).(c) Comparison of nuclear volumes after segmentation of plant nuclei by the Otsu or gift-wrapping methods. Nuclei were split into two categories: guard cells (n = 375) and pavement cells (n = 127) (Supplemental table 4) were segmented by the two methods and volumes of the segmented nuclei were computed by NucleusJ 2.0. Modified Otsu method (gray); gift-wrapping (green). Student t-test P-value: *** <0.0001, * = 0.0046.(d) Principal component analysis of morphology parameters (Flatness, Elongation, New surface area and Volume) obtained after segmentation by the gift-wrapping (left) or Otsu (right) methods of the same nuclei as in Figure 2c. Guard cell nuclei (GC, black) and pavement cell nuclei (PC, orange).

Careful analysis identified two main characteristics of the nuclei that were poorly segmented. First, the nucleolus that is not stained by DNA dyes such as DAPI or Hoechst is often excluded during the segmentation step. This phenomenon is common for large nuclei containing large nucleoli. A simple fill step was then introduced before segmentation. However, this did not solve the abnormal segmentation when the nucleolus is close to the nuclear periphery (Figure 2(a)). Second, very high signal intensity was observed for some chromocenters of large nuclei leading to the detection of chromocenters not as an object within a nucleus but as a distinct object (*i.e*. a nucleus). This results in segmentation of the nucleus into several small objects. In summary, poor segmentation of plant nuclei was strongly biased toward larger nuclei with high contrast between chromocenters and nucleolus that generated concavity artifacts and holes. This also induced a bias in our analysis as a substantial fraction of the larger nuclei was discarded from the analysis.

To improve our 3D segmentation process, an alternative method, hereafter called gift-wrapping, was implemented in 3D using an edge-based segmentation [37]. The algorithm segments one 2D slice at once in all orientations of the 3D object before building the 3D segmentation (Materials and Methods).

The gift-wrapping method was implemented in NucleusJ 2.0, which now produces three folders *i.e*. one folder for nuclei segmented by the Otsu modified method, one segmented by the gift-wrapping method and a third one containing the nuclei that cannot be segmented. This last category is classified as ‘bad crop’ (Supplemental table 3). First, we confirmed that the method did not alter the segmentation of small convex objects with a homogeneous signal, like microspheres (Figure 2(b), Supplemental table 2). Then, a dataset of 502 nuclei from WT plants stained with Hoechst was used to evaluate the appropriateness of the method (Supplemental table 4). Shape artifacts due to the nucleolus or to low staining intensity were efficiently removed (Figure 2(a)). The limit of this method is its accuracy, as the measured nuclear volume is significantly increased especially for small objects such as guard cell nuclei (Figure 2(c)). This over-segmentation also leads to the loss of 0.02% of the nuclei (Supplemental table 4) located at the boundaries of the 3D images that are then considered as incomplete by the software. Although the gift-wrapping method increases the size of segmented objects, PCA analysis confirmed that it does not introduce bias in the analysis and that the two nuclei populations of different cellular origin are still nicely distinguished (Figure 2(d)).

The 3D gift-wrapping segmentation process therefore resolves some drawbacks of our previous Otsu modified method by decreasing the bias previously observed for the segmentation of larger nuclei. It provides an alternative, more efficient, segmentation method for plant nuclei.

### Computer parameter optimization

Describing nuclear morphology requires an accurate estimation of the nuclear volume and surface area. NucleusJ 1.0 takes advantage of MorphoLibJ, a library that calculates the volume as the sum of voxels included into the segmented nucleus, multiplied by the volume of an individual voxel (voxel calibration) and computes the surface area through a modified version of the Crofton formula [36].

**Figure 3.**
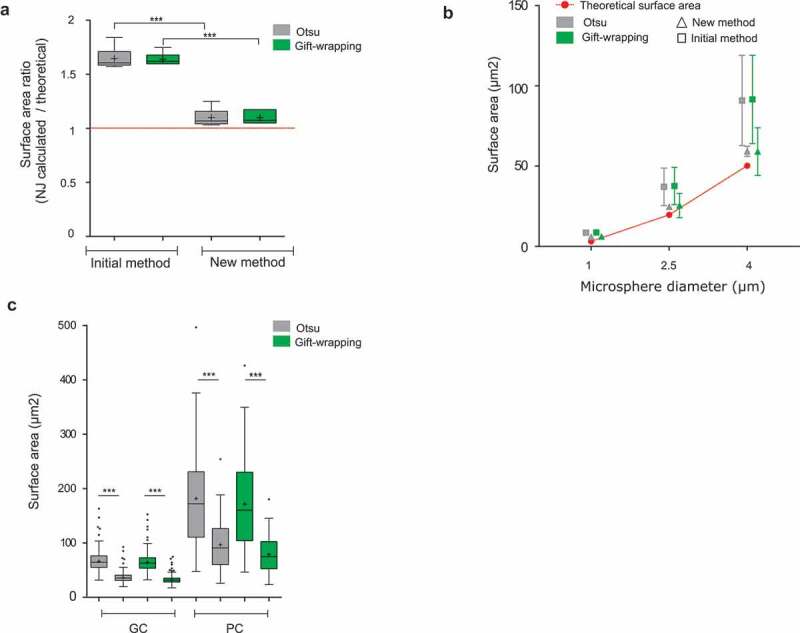
Evaluation of a new method of Surface Area calculation implemented into NucleusJ 2.0

To compute the surface area, surfaces of all voxels delimiting the boundary of the object are summed [23]. However, this calculation was the more error-prone, with the error increasing for small-segmented objects consisting of only a few voxels. This approximation led to significant over-estimation of the surface area, when artificial objects or standardized microspheres were measured with NucleusJ 1.0 (Figure 3(a)).

(a) Digitized spheres of various radii of 5, 10, 20, 30, 40, and 50 voxels were generated and used to calculate the surface area (Supplemental table 1) with the initial and newly developed calculation method. Data are presented as a ratio between the observed and theoretical size of the digitized spheres. Student t-test P-value: *** <0.0001.(b) Fluorescent Microspheres of diameter of 1, 2.5, and 4 µm (n = 28, 24, and 15, respectively) were imaged using the wide-field microscope with an optigrid module, subjected to autocrop and segmented by the two NucleusJ 2.0 segmentation methods. Surface area gained from Otsu and gift-wrapping segmentation was then computed with the initial and new method of calculation (Supplemental table2). Red: theoretical surface area; gray: Otsu method; green: gift-wrapping method; triangle: new method; square: initial method. (c) Plant nuclei. Surface area of nuclei from cotyledon epidermis of WT plants were segmented by the Otsu or gift-wrapping methods. Nuclei from guard cells (GC, n = 375) and pavement cells (PC, n = 127) (Supplemental table 4). Student t-test P-value: *** <0.0001.

To correct the surface area computation, a new method (Materials and Methods) was implemented in NucleusJ 2.0 to calculate the surface element (surfel) contribution of each voxel using an iterative convolution method based on *on-surface convolution* [38]. The algorithm browses each boundary voxel delimiting the 3D object from the background of the segmented image. For each boundary voxel, the contribution of each surfel to the final area is computed. While the surface area calculation remains imperfect, it gives more meaningful results for digitized spheres of variable radius with a ratio between calculated and theoretical surface area of 1.068 and 1.084 instead of 1.608 and 1.636, respectively, for the Otsu and gift-wrapping segmentation methods (Figure 3(a)). The accuracy of our surface calculation was also assessed using standardized microspheres of three distinct diameters ranging from 1 to 4 µm. For these, the new surface area calculation was closer to the theoretical values (Figure 3(b)). Surface area was also assessed for a dataset of 502 nuclei from WT plants stained with Hoechst indicating that the surface area was over-estimated when the surface area was calculated with NucleusJ 1.0 (Figure 3(c)).

Taken together, a new surface area calculation is now introduced in NucleusJ 2.0 to obtain a more realistic value of this key parameter.

### Application of NucleusJ 2.0 to quantify alterations of nuclear morphology and nuclear organization

To better understand the contribution of nuclear morphology to chromatin organization, one possible approach is to compare WT and mutant plants. *crwn* mutants are well described and show altered nuclear morphology, chromatin organization and gene expression [7–10]. *CRWNs* are a small gene family composed of four members with CRWN1 proteins having major effects on nuclear morphology (smaller and rounder nuclei in *crwn1* mutants) and CRWN4 having major effects on chromatin organization (dispersed chromocenters and 5S rRNA gene signals in *crwn4* mutants). Additive phenotypes are observed in the double *crwn1 crwn4* mutant [8]. Furthermore, an additional protein of the plant nuclear lamina called KAKU4 was found to co-immunoprecipitate with CRWN1 and CRWN4 [11].

**Figure 4.**
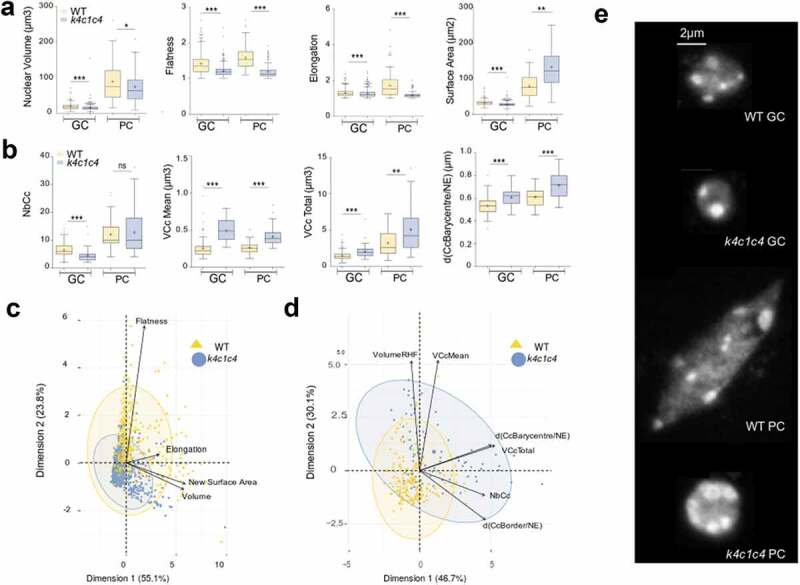
NucleusJ2.0 analysis of the *k4c1c4* mutant with 1altered nuclear morphology and chromatin organization

**Figure 5.**
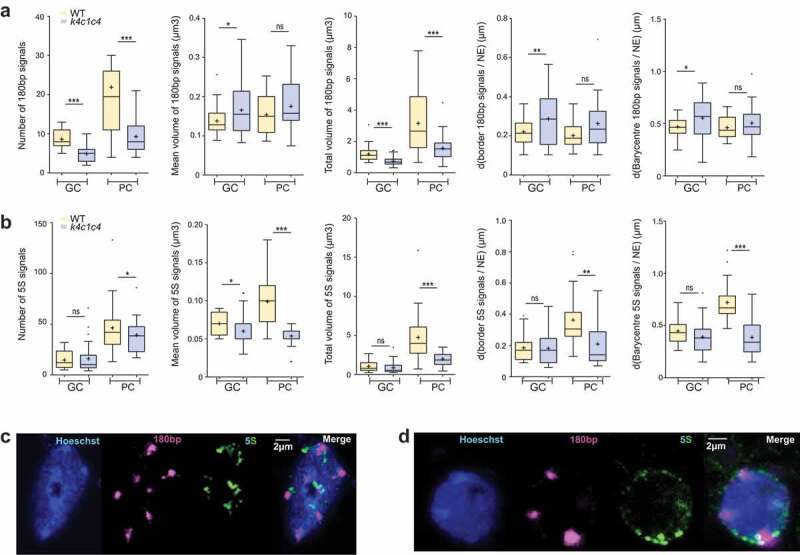
Analysis of aspect and position of 180pb and 5S rDNA repeats revealed by 3D-DNA FISH using NucleusJ 2.0

We, therefore, used NucleusJ 2.0 to compare WT *Col-*0 seedlings (hereafter WT) and a *kaku4-2 crwn 1–2 crwn 4–1* mutant in the *Col-*0 genetic background (hereafter called *k4c1c4* mutant). The *k4c1c4* mutant was chosen for its strong impact on plant growth (Supplemental Figure 1(a)). We acquired 35 and 28 wide-field stacks, respectively, from 12 WT and 10 mutant plants stained with Hoechst. After the autocrop and segmentation process, WT (n = 502) and *k4c1c4* mutant (n = 672) nuclei were annotated manually as guard (GC) or pavement cell (PC) nuclei after examination of the Z-projection (Supplemental tables 3–4). Despite the reduced plant size of the triple mutant, we found an increased number of stomates (7.5 and 10.11, respectively, in WT and *k4c1c4* mutant; Supplemental table 5) and observed a significant increase in nuclear density both in guard and pavement cells (Supplemental Figure 1b). The dataset was then used to assess nuclear morphology and chromocenter organization. Results gained from our new gift-wrapping method are presented in Figure 4. The nuclei of the *k4c1c4* mutant are strongly affected for most of the nuclear parameters and show reduced nuclear size, reduced elongation (Figure 4(a)) and fusion of chromocenters leading to an increased chromocenter volume (Figure 4(b)). This is very similar to previous results obtained with *crwn1 crwn2* mutant [23]. NucleusJ can also be used to quantify 3D DNA FISH signals. To illustrate this application, two new datasets of 3D-DNA-FISH images were used to investigate the effect of the triple *k4c1c4* mutant. The distinct fluorescent channels were collected corresponding to signals from the DNA dye and the probes for 180bp and 5S rDNA repeats (Supplemental tables 7–8). The *k4c1c4* mutant shows a reduced number of 180bp signals, however, situated at similar positions close to the nuclear periphery as in WT nuclei (Figure 5(a) and 5(c)). In contrast, 5S rDNA loci, while their number is very similar to the WT, are smaller in size and localize closer to the nuclear envelope (Figure 5(b) and 5(d)). Each of the NucleusJ 2.0 parameters describing the *k4c1c4* mutant is illustrated in Supplementary Figure 2 indicating a very strong effect of the mutant background on the organization of the 180bp satellite repeats.

Nuclear morphology parameters were computed by the gift-wrapping segmentation method using an initial dataset of WT (n = 663) and *k4c1c4* mutant (n = 881) nuclei described in Supplemental table 4.(a) Nuclear morphology parameters computed by NucleusJ 2.0 on GC and PC cells: nuclear volume (µm^3^), flatness, elongation and surface area (µm^2^). WT (n = 502) and *k4c1c4* mutant (n = 672) (Supplemental table 4). Student t-test P-value: * <0.01, ** <0.001 and *** <0.0001.(b) Chromatin organization parameters computed by NucleusJ 2.0: number of chromocentres (NbCc), Mean of chromocentre volume (VCc Mean, µm^3^), total volume of chromocentres (VCc Total, µm^3^), and distance between chromocentre barycenter and the nuclear envelope (d(CcBarycentre/NE), µm). WT (n = 186) and *k4c1c4* mutant (n = 81) (Supplemental table 6). Student t-test P-value: ns > 0.01, ** <0.001 and *** <0.0001.(c) and d) Principal component analysis using morphological parameters (n = 1544; Supplemental table 4) and chromatin organization parameters (n = 267; Supplemental table 6).(e) Typical images chosen with parameters close to the median values of morphological parameters and chromatin organization parameters. Z-projection of raw nuclei. Scale Bar 2 µm. GC: guard cell, PC: pavement cell.

NucleusJ 2.0 parameters applied to A) 180bp signals and B) 5S rDNA. Parameters: number of DNA FISH signals, mean volume of FISH signal (µm^3^), total volume of FISH signal (µm^3^), distance between FISH signal border and the nuclear envelope (d(FISH signal Border/NE), µm) and distance between FISH signal barycenter and the nuclear envelope (d(Barycenter of FISH signal/NE), µm) (Supplemental tables 7–8). Student t-test P-value: ns >0.01, * <0.01, ** <0.001 and *** <0.0001. C) Typical 3D DNA FISH Z-projection of pavement cell nuclei of WT (n = 65 for 180bp and n = 32 for 5S) and D) *k4c1c4* mutant (n = 95 for 180bp and n = 48 for 5S) (Supplemental tables 7–8). From left to right: Hoechst (DNA, blue), 5ʹ TYE 563 LNA probe (180bp, purple), CY5 PCR probe (5S, green) and merge. Scale Bar 2 µm.

In summary, we demonstrate that the new version of NucleusJ 2.0 can also be efficiently used to quantify alterations of nuclear domains as revealed by DNA dyes (*i.e*. chromocenters) or by 3D-DNA FISH (180bp satellite repeat and 5S rDNA arrays).

## Discussion

Nuclei morphology is intrinsically linked to biological processes like gene regulation or development and has been widely used as a marker for human disease [21]. Microscopy imaging provides a classical mean to investigate nuclear morphology variations with segmentation as a critical step in the image analysis process. Although large numbers of segmentation methods are available, no universal segmentation technique will work for all images and all model systems and only few of them are suitable for big data analyses.

We provide evidence that our methods to calculate nuclear morphology parameters are accurate by using virtual objects like digitized spheres and standardized microbeads usually used to calibrate confocal microscopes and cell sorters.

The first step in NucleusJ is to describe the nuclear morphology. We have now implemented two complementary segmentation methods into NucleusJ 2.0 that give slightly different results. As a simple and fast method, the Otsu method relies on the application of a threshold to distinguish the object from the background, but defining the most relevant threshold for each single nucleus of a wide-field stack is not a simple task as local variations have to be taken into consideration. This method is also sensitive to nuclear indentations and low labeling area at the position of the nucleolus often located close to the nuclear periphery. The gift-wrapping method was shown to be less sensitive to these factors. It has however the drawback of slightly increasing the object volume and it requires more computing resources than the Otsu method. This is one of the reasons to offer a command line version of NucleusJ 2.0 to use more powerful servers at computing centers. Taken together, the two methods are considered complementary to each other and present two possible alternatives to generate segmented datasets.

The autocrop process will contribute to middle throughput 3D image analysis. It automatically produces an average of 21 isolated 3D nuclei in about 6 minutes reducing substantially the time-intensive manual process. Starting from these large populations of nuclei, NucleusJ 2.0 then describes automatically the 3D nuclear morphology.

The second step in NucleusJ 2.0 was to describe chromatin organization and a triple *kaku4-2 crwn 1–2 crwn 4–1* mutant was used to illustrate the potential of our new software. In the triple mutant some nuclear morphology parameters are altered: guard cells show reduced nuclear volume, while pavement cells show an increased surface area and both types of nuclei revealed reduced flatness in the mutant background.

We illustrated the use of NucleusJ 2.0 to measure FISH signals in a quantitative manner in order to estimate the volume occupied by the FISH signals. Then, the border or the barycenter of the signal are used to compute the positions of the FISH signal in respect to the nuclear periphery. This offers an original method to quantify chromatin compaction while most softwares often define FISH signals as standardized spots that can only be used to compute distances. Using this method we observed that the 180bp signals are more dispersed while the 5S rDNA loci seem more condensed in the mutant background and preferentially locate at the nuclear periphery. All these parameters were detected successfully by the new software update and are consistent with previous observations [8].

Segmentation of nuclear domains remains challenging to characterize nuclear architecture and NucleusJ 2.0 can provide a solution. In future, further optimization of nuclear domain detection and quantification are required as this still relies on a 3D watershed process, which is time-consuming and user-dependent. Recent progress in Artificial Intelligence is opening up new opportunities to improve 3D bio-imaging. Convolutional Neural Networks (CNN) such as those developed for U-Net, Ilastik, or Star dist [45–47] have been successfully applied for 3D bio-imaging. For the 3D watershed process, preliminary tests using a U-Net CNN [46] currently under development are promising. We expect that the present study, which provides several well-annotated training datasets, will help to pave the way for the development of CNN methods, which we expect will transform the way we acquire and analyze 3D images creating an automated high-performance tool well designed for big-data analysis. Here it is important to highlight the need for large, high-quality datasets to train new CNNs. When looking at image repositories, large-annotated 3D nuclear datasets, digitized objects or standardized microspheres are not easily accessible to benchmark new software or train new networks. This work supported by the COST-Action CA162121 is a first attempt to provide such datasets to the community through a fully public repository with free access to the data.

## Supplementary Material

Supplemental Material
