# Supplementary material for: Automated 3D bio-imaging analysis of nuclear organization by NucleusJ 2.0
Source: Nucleus. 2020 Nov 29;11(1):315–29. doi: 10.1080/19491034.2020.1845012 (PMC7714466; doi:10.1080/19491034.2020.1845012)
Supplement: Supplemental Material [file KNCL_A_1845012_SM0256.zip › Supplementary information/Supplemental files1-3.docx]

**Supplemental file 1: Autocrop**

**Introduction :**

To extract large numbers of nuclei from a wide-field image, we have designed a process called *autocrop* here performed in 3D although this can also be achieved for 2D images. The basic principles of the autocrop process rely on a simple Otsu threshold method applied to the wide-field image. bounding box surrounding the 3D nuclei are defined to crop the nuclei from the wide-field image. To avoid to select too many objects which are not nuclei, a simple and scalable size filter was introduced. A second scalable filter allows to limit the number of multiple boxes for a given nucleus especially for large nuclei. Autocrop parameters are set by default but can be modified prior to the analysis through a configuration file (*config file*).

**Launch :**

- Command line

1. Go into your terminal
2. Enter : java -jar NucleusJ_2.jar autocrop *input* *output config_file*

- ImageJ (Fiji)
  1. Add the .jar file into the plugins folder of ImageJ.
  2. Go to plugins -> install and select the jar file.
  3. Restart ImageJ.
  4. Go to plugins -> NucleusJ -> Autocrop NucleusJ 2.
  5. Enter or Select the *input* and *output.*
  6. Choose to use the default configuration, modify the parameters, or enter/select the *config_file.*
  7. Click “Start” to begin the Autocrop on the *input*.

**Input :**

- Input folder/file

Path to the directory that contains the wide fields to crop.

Works with 2D or 3D wide field files.

List of current formats tested : TIF , CZI , ND.

Giving a single file as “Input directory” will works.

- Output folder

Path to the directory that will contains the results of the Autocrop.

NucleusJ will create the output directory if it can’t find it.

- Config_file (optional)

Use it to set calibration parameters manually following this format:

“Parameter name”: “value” (one per line)

- Parameters

They can be found in the AutocropParameters class and can be set through the config file or the class itself by passing the parameters through its constructor:

| Parameter name | Default value | Value type | config file | Description |
| --- | --- | --- | --- | --- |
| Gaussian Blur (x,y,z) | 0.5, 0.5, 1 | double | false | Gaussian blur’s parameters |
| xCropBoxSize | 40 | Int | True | Number of voxels added to xmin and xmax of the connected component defining the final box size in x |
| yCropBoxSize | 40 | Int | True | Number of voxels added to ymin and ymax of the connected component defining the final box size in y |
| zCropBoxSize | 20 | int | True | Number of voxels added to zmin and zmax of the  connected component defining the final box size  in z |
| minVolumeNucleus | 1 | Int | True | Minimum volume of detected object |
| maxVolumeNucleus | 2147483647 | Int | True | Maximum volume of detected object |
| thresholdOSTUcomputing | 20 | Int | True | Minimal default OTSU threshold |
| channelToComputeThreshold | 0 | int | True | Channel number used to compute OTSU threshold (Channel 1 is 0 etc) |
| slicesOTSUcomputing | 0 | Int | True | Slice start used to compute OTSU threshold |
| boxesPercentSurfaceToFilter | 50 | Int | True | Surface percent of boxes to groups them |
| boxesRegroupement | true | boolean | true | Activation of boxes regroupement |
| xcal | 1 | Double | True | X calibration value |
| ycal | 1 | Double | True | Y calibration value |
| zcal | 1 | Double | True | Z calibration value |

**Output :**

The output directory will contain all cropped images and a result_Autocrop_Analyse.csv file with all information about the process like parameters and results.

In result_Autocrop_Analyse.csv, you will find:

- When the process started (YYYY-MM-DD:HH-mm-ss).
- The input and output folders.
- The calibration.
- Process parameters values (listed above in Input parameters section).
- And then, a list of characteristics for each one of the segmented images:
  1. Nucleus file name
  2. Number of crops
  3. OTSU threshold

Default OTSU threshold.

**Supplemental file 2: 3D gift-wrapping**

To better define the edge of the object, and delete shape irregularities generated by the initial step of segmentation, a 3D gift-wrapping method was implemented in NucleusJ 2.0.

This approach is neither a convex hull, nor an approximation of a convex hull, nor a simplified convex hull, but a new notion, which can be named 3D gift-wrapping of a set of voxels. The idea is that, due to *a priori* knowledge from biology, and due to the goal of this study which is to improve the geometric accuracy of the segmented nucleus, the nucleus cannot be considered as a convex object. The nuclear shape is essentially non-convex, but the aim is to remove some artifactual concavities, while preserving the natural ones, using a relevant distance threshold in the 3D gift-wrapping algorithm.

For the sake of simplicity, this method was designed in 2D and then implemented slice by slice up to the final 3D volume. Hence, depending on the axis used to decompose the volume into slices, three different volumes can be obtained. Then the union of the three volumes was built.

In each slice, in order to tune the 3D gift-wrapping algorithm, and to fill the shape artefacts, a parameter of maximal threshold distance *td* was applied between two vertices which will defined the final boundary. The best threshold distance *td* was determined experimentally as the half of the estimated radius of a sphere with a volume equivalent to the object one:

$$td=\frac{\sqrt[3]{\frac{3Volume}{4}}}{2}$$

The method of threshold distance efficiently removes shape artefacts, although keeping real indentations occurring at the nuclear surface. As the developed algorithm takes into account only one slice at a time, several connected components might appear on certain slices (for example due to the artifactual shapes of the objects). If several objects are detected on a given slice, the connected components are labeled and then the 3D gift-wrapping algorithm is applied for each label.

**3D gift-wrapping algorithm**

The algorithm is applied slice by slice through the objects. Let’s now consider a given slice, *i.e.* a 2D plane, where a set of boundary voxels is located. Our goal is to apply our modified 2D gift-wrapping algorithm on this plane.

The usual 2D gift-wrapping algorithm starts by defining an initial vertex $v_{n}$. In our implementation, $v_{n}$ is defined as the closest boundary voxel to the top edge of the image. To find the next voxel belonging to the hull, a loop browses through every other boundary voxel $v_{b}$ in the image that does not yet belong to the hull.

In the usual 2D gift-wrapping method, a voxel is selected to belong to the hull if all the other boundary voxels are located to the right of the vector $\vec{V_{current}}$ = $v_{b}$-$v_{n}$=$\left( v_{b}x-v_{n}x,v_{b}y-v_{n}y \right)$. But, as we would like to preserve the shape artefacts, $\vec{V_{current}}$ is submitted to the additional condition that its norm should be lower than the previously defined threshold *td*. Given a voxel $v_{n}$, the search space of the algorithm finding the ideal voxel $v_{b}$ is thus limited to a disc of center $v_{n}$ and radius *td*.

Under such a condition the usual 2D gift-wrapping method cannot be applied, as, in some cases, it is impossible to find a point in the previous disc that verifies the property: “all the boundary voxels are located to the right of the vector $\vec{V_{current}}$”. To solve this problem, we consider the oriented angle $\alpha\in]-, ]$ between $\vec{V_{current}}$ and the previous value of $\vec{V_{current}}$ named $\vec{V_{test}}$. $\vec{V_{test}}$ is initialized with the default value of (10,0), a horizontal vector along the $\vec{x}$ axis, which does not belong to the envelop due to the definition of the initial value of $v_{n}$. The goal then is to find $v_{b}$ such that α is minimal. When α has been determined the loop restarts until the whole hull is completed (until the initial voxel is reached).

**Supplemental file 3: Nuclear Morphology**

**Introduction :**

NucleusJ a simple and user-friendly ImageJ plugin dedicated to the characterisation of nuclear morphology and chromatin organisation in 3D. NucleusJ quantifies parameters including shape and size of nuclei as well as intranuclear objects and their position within the nucleus. This documentation describes characteristics sorted by the nuclear segmentation step of NucleuJ 2.0. 19 characteristics are produced and listed in this documentation. The documentation explains how to **launch** the plugin, how to call the **input** dataset and produce **output** files. Parameters of the plugin are scalable through a configuration file (**config file**).

NucleusJ 2.0 includes two different segmentation methods hereafter named Otsu modified method and gift-wrapping method.

**Launch :**

- Command line

1. Go into your terminal
2. Enter : java -jar NucleusJ_2.jar segmentation *input* *output config_file*

- ImageJ (Fiji)
  1. Add the .jar file into the plugins folder of ImageJ.
  2. Go to plugins -> install and select the jar file.
  3. Restart ImageJ.
  4. Go to plugins -> NucleusJ -> Segmentation NucleusJ 2.
  5. Enter or Select the *input* and *output.*
  6. Choose to use the default configuration, modify the parameters, or enter/select the *config_file.*
  7. Click “Start” to begin the Segmentation on the *input*.

**Input :**

- Input folder/file

Path to the directory that contains the cropped images.

Works with 2D or 3D image files.

Giving a single file as “Input directory” will works.

- Output folder

Path to the directory that will contains the results of the Segmentation.

NucleusJ will create the output directory if it can’t find it.

- Config file (optional)

Use it to set calibration parameters manually following this format:

“Parameter name”: “value” (one per line)

- Parameters

They can be found in the SegmentationParameters class and can be set through the config file or the class itself by passing the parameters through its constructor:

| Parameter name | Default value | Value type | config file | Description |
| --- | --- | --- | --- | --- |
| giftWrapping | true | Boolean | True | Is gift wrapping enabled |
| minVolumeNucleus | 1 | Int | True | Minimum volume of detected object |
| maxVolumeNucleus | 3000000 | Int | True | Maximum volume of detected object |
| xcal | 1 | Double | True | X calibration value |
| ycal | 1 | Double | True | Y calibration value |
| zcal | 1 | Double | True | Z calibration value |

**Output :**

The output directory contains all segmented images and a result_Segmentation_Analyse.csv file with all information about the process like parameters and results, all in 2 directories called OTSU and GIFT which are different segmentation methods. There will be 2 versions of each segmented images, in each directory.

In result_Segmentation_Analyse.csv, you will find:

- When the process started (YYYY-MM-DD:HH-mm-ss).
- The input and output folders.
- Process parameters values (listed above in Input parameters section).
- And then, a list of characteristics for each one of the segmented images:
  1. Nucleus file name
  2. Volume
  3. Flatness
  4. Elongation
  5. Sphericity
  6. Esr (Equivalent Sphere Radius)
  7. Surface area
  8. Surface area corrected
  9. Sphericity corrected
  10. Mean intensity nucleus
  11. Mean intensity background
  12. Standard deviation
  13. Min intensity
  14. Max intensity
  15. Median intensity image
  16. Median intensity nucleus
  17. Median intensity background
  18. Image size
  19. OTSU threshold
