# Supplementary material for: Automated 3D bio-imaging analysis of nuclear organization by NucleusJ 2.0
Source: Nucleus. 2020 Nov 29;11(1):315–29. doi: 10.1080/19491034.2020.1845012 (PMC7714466; doi:10.1080/19491034.2020.1845012)

**Supplemental table 1: Digitised spheres datasets**

Spheres of radius of 5, 10, 20, 30, 40 and 50 isotropic voxels (X, Y, Z = 1, 1, 1) were generated in ImageJ.

**A) Volumes of theoretical spheres** were calculated according to the following formula: 4/3π x radius^3^. Volumes were estimated by segmentation of the digitised spheres using our two methods of segmentation (modified Otsu and gift-wrapping) and the ratio between NucleusJ 2.0 computed value/theoretical value was calculated. P-Value of Student t-tests between theoretical versus Otsu, theoretical versus gift-wrapping and Otsu versus gift-wrapping are respectively 0.1163, 0.1365 and 0.02022.

**B) Surface areas of theoretical spheres** were calculated according to the following formula: 4π x radius^2^. Digitised spheres were segmented by the two methods of segmentation (modified Otsu and Gift-wrapping) and surface area calculation was performed using the initial (NucleusJ 1.0) and new (NucleusJ 2.0) method of calculation. Ratio between NucleusJ 2.0 computed value/theoretical value was calculated. These results were used to construct **Fig. 3A**.


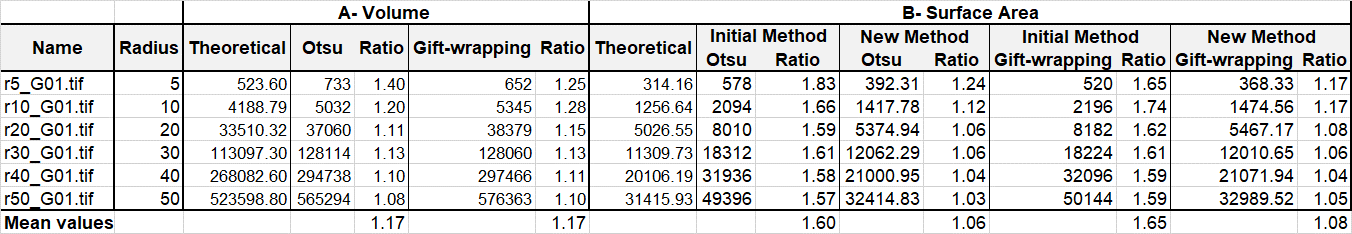


**Supplemental table 2: Fluorescent microsphere dataset.**

**1- Volumes (used in Figure 2B)**

**A)** Fluorescent microspheres of 1, 2.5 and 4µm diameters were used.

**B)** Theoretical volume was calculated according to the following formula: 4/3 π x radius^3^.

**C)** Number of Wide-field stacks and cropped images for DAPI and FITC fluorescent channels.

**D) E)** Wide-field stacks were acquired with a MMAF+Optigrid microscope using a x63 oil objectives. DAPI and FITC fluorescent channels were used to generate the autocrops. Autocrops were processed by NucleusJ 2.0 to compute the microsphere volumes (volume) expressed in µm^3^ using the 3D gift-wrapping (**D**) or modified Otsu (**E**) methods. Standard deviation (SD) and number of microsphere images (N) are given. To construct **Fig. 2B**., results from the DAPI and FITC channels were merged to obtain the table below.


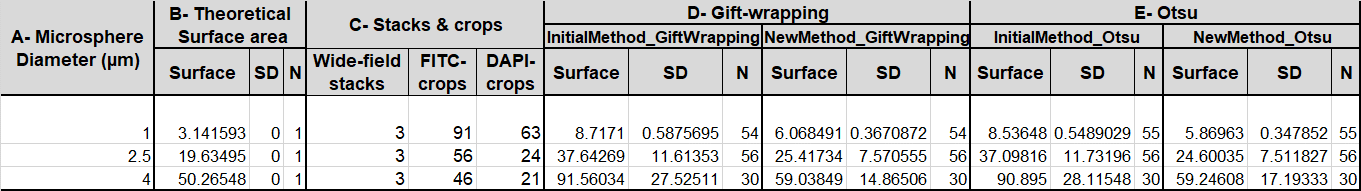


**2- Surfaces (used in Figure 3B)**

**A)** Fluorescent microspheres of 1, 2.5 and 4µm diameters were used.

**B)** Theoretical surface was calculated according to the following formula: 4π x radius^2^.

**C) D)** Wide-field stacks were acquired with a MMAF+Optigrid microscope using a x100 or a x63 oil objectives. DAPI and FITC fluorescent channels were used to generate the autocrops. Autocrops were processed with 3D gift-wrapping (**C**) and modified Otsu (**D**) methods respectively using NucleusJ 1.0 (InitialMethod) or NucleusJ 2.0 (NewMethod). The microsphere surfaces (surface) expressed in µm^2^. Standard deviation (SD) and number of microsphere images (N) are given. To construct **Fig. 3B.,** results from the DAPI and FITC channels were merged to obtain the table below.


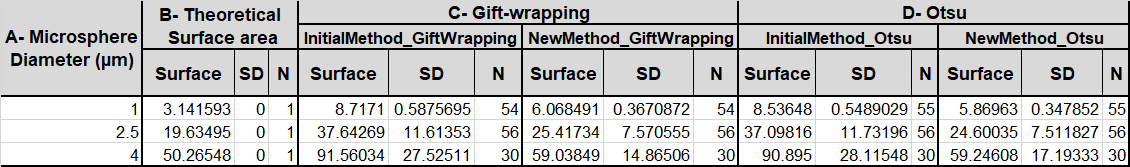


**Supplemental table 3: Evaluation of autocrop efficiency using a WT (Col-0) dataset.**

As a standard procedure, 8-12 cotyledons were used in a typical experiment. Three image stacks of 210µmx210µm and 75 Z-sections were usually collected for each cotyledon. This dataset was used to illustrate the efficiency of the autocrop process. As wide-field stacks of plant cotyledon contain multiple nuclei, an autocrop process was used to isolate a collection of thumbnails containing one nucleus per image. Manual inspection of the maximal Z-projections of the 694 remaining crops scored 92 nuclei that were damage or having a low signal versus noise ratio or were not a nucleus (abnormal crop). In subsequent segmentation steps by NucleusJ 2.0, crops containing incomplete nuclei are automatically discarded and the corresponding raw files are moved in a “bad crop” folder.


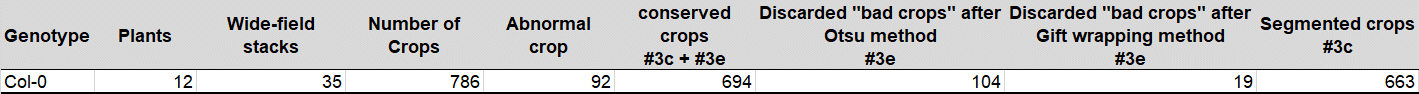


**Supplemental table 4: WT and *k4c1c4* mutant** **datasets for nuclear morphology characterisation.**

The number of plants, number of wide-field stacks, crops, guard cell (GC) and pavement cell (PC) nuclei are given as well as the number of discarded crops after segmentation (abnormal crops - described in Supplemental table 3). Only 16 (11+5) abnormal segmented nuclei corresponding to close merged nuclei or to a nucleus merged with a noise artefact (abnormal gift-wrapping segmentation) were recorded by visual inspection of 229 nuclei with a ratio volume gift-wrapping/Otsu >1.2. These 16 nuclei were included in the analysis.

WT dataset was used to generate **Fig. 2C** and **3C**. WT and *k4c1c4* for Fig.**4A**, **4B**,


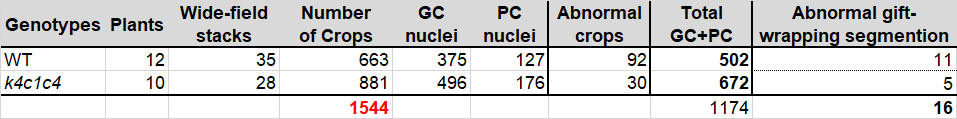


**Supplemental table 5: Characterisation of the density of guard cells (GC), pavement cells (PC) nuclei and stomates per image in WT and *k4c1c4* mutant.**

Number of original wide-field stacks, total number of GC and PC (similar to those of Supplemental table 4) and mean value of the number of stomates per image are given at the bottom of the table.


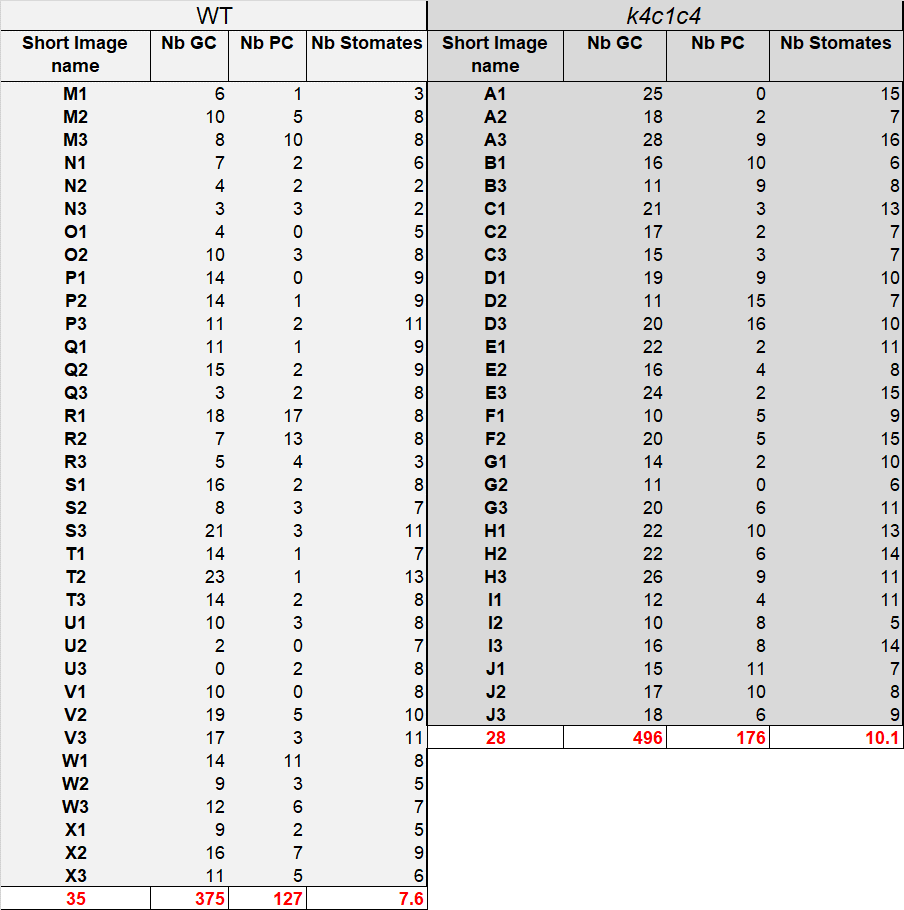


**Supplemental table 6: WT and *k4c1c4* mutant datasets for characterisation of chromocentre organisation.**

The number of plants, number of wide-field stacks, crops, guard cells (GC) and pavement cells (PC) nuclei are given. Note that although the original 3D images are similar to those of Supplemental Table 4, only a fraction of the nuclei were subsequently segmented through the 3D watershed to segment the chromocentres. The 3D watershed step still requires a manual selection of the most relevant threshold to be applied. 215/502 (WT) and 100/672 (mutant) were segmented for this analysis.


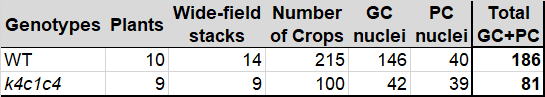


**Supplemental table 7: WT and *k4c1c4* mutant datasets for characterisation of 180 pb DNA FISH signals.**

The number of plants, number of wide-field stacks, crops, guard cell (GC) and pavement cell (PC) nuclei are given.


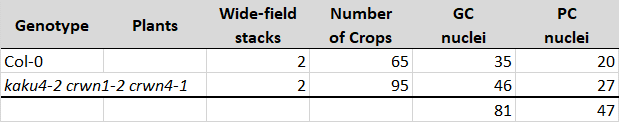


**Supplemental table 8: WT and *k4c1c4* mutant datasets for characterisation of 5S DNA FISH signals.**

The number of plants, confocal images, guard cells (GC) and pavement cells (PC) nuclei are given.


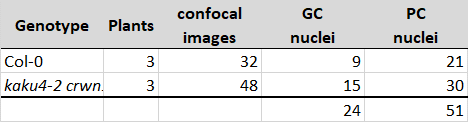

Supplement: Supplemental Material [file KNCL_A_1845012_SM0256.zip › Supplementary information/Supplemental tables 1-8_CLEAN.docx]
